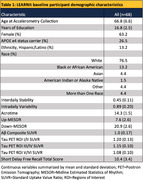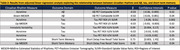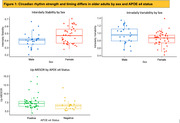# Advanced circadian rhythms in older adults are associated with higher amyloid‐β and tau PET burden and poorer short‐term memory

**DOI:** 10.1002/alz.089962

**Published:** 2025-01-09

**Authors:** Joanna L Eckhardt, A. Lisette Isenberg, Joy Stradford, Vahan Aslanyan, Laura E. Fenton, Teresa Monreal, Judy Pa

**Affiliations:** ^1^ Neurosciences Graduate Program, University of California, San Diego, La Jolla, CA USA; ^2^ Alzheimer's Disease Cooperative Study (ADCS), University of California, San Diego, La Jolla, CA USA; ^3^ University of Southern California, Los Angeles, CA USA

## Abstract

**Background:**

Several studies link circadian rhythm disturbances to Alzheimer's disease. However, little is known about circadian rhythm involvement with Alzheimer’s pathology in early stages of the disease. The current study investigates the relationship between circadian rhythms, Aβ and tau PET, and short‐term memory, and explores how circadian rhythms vary between age, sex, and APOE4 status.

**Method:**

This research utilizes baseline data from the Lifestyle Enriching Activities for Research in Neuroscience Intervention Trial. Sixty‐eight older adults ages 55‐80 experiencing early cognitive changes wore a GENEActiv accelerometer for ∼30 days. Accelerometer data was processed using the R package GGIR (v3.0‐1), and circadian rhythm measures were extracted using nparACT (v0.8) and ActCR (v0.3.0). Circadian rhythm variables include interdaily stability (IS) (rhythm synchronization), intradaily variability (IV) (fragmentation), and acrotime (peak activity time). Up‐MESOR and Down‐MESOR signify timing of activity increasing above or below mean activity level, respectively, with lower values indicative of advanced (earlier) circadian phase. Participant Aβ and tau SUVr were measured using PET imaging. Short‐term memory is measured with the California Verbal Learning Test‐II Short Delay Free Recall Total Score. Linear regression models were conducted to test associations between circadian rhythms, Aβ, tau, and short‐term memory, and were adjusted for age, sex, APOE4 status, and years of education. Pearson’s correlation was conducted between circadian rhythms and age. Wilcoxon Rank Sum tests were used to assess differences in circadian rhythms between sex and APOE4 status.

**Result:**

Participant demographics are summarized in Table 1. Regression results are summarized in Table 2. Acrotime and Down‐MESOR were associated with Aβ Composite. Acrotime and Up‐MESOR were associated with tau across multiple Braak regions of interest. Notably, IV was significantly associated with Braak stage III/IV. Both acrotime and Up‐MESOR were associated with Short Delay Free Recall Score. Acrotime (r =‐0.25, p=0.04) and Down‐MESOR (r=‐0.26, p=0.04) were moderately correlated with age. IS (W=368, p=0.03) and IV (W=694, p=0.05) differed by sex, and Up‐MESOR differed by APOE4 status (W=599, p=0.03) (Figure 1).

**Conclusion:**

Participants with advanced (earlier) circadian rhythms demonstrate higher Aβ and tau PET burden and poorer short‐term memory. Circadian rhythm measures vary with age, sex, and APOE4 status.